# Effect of Ca and Zr Additions on Microstructure and Mechanical Properties of As-Extruded Mg-3Sn Alloy

**DOI:** 10.3390/ma15186343

**Published:** 2022-09-13

**Authors:** Zheng Jia, Yongzhi Yu, Bing Yu, Li Fu, Wenyi Hu, Yichuan Shao

**Affiliations:** 1 College of Mechanical Engineering, Shenyang University, Shenyang 110044, China; 2 College of Chemistry and Materials, Longyan University, Longyan 364012, China; 3 College of Intelligent Science and Engineering, Shenyang University, Shenyang 110044, China

**Keywords:** magnesium alloys, mechanical properties, grain refinement, CaMgSn phase

## Abstract

In this paper, the effect of Ca and Zr additions on microstructure and mechanical properties at room temperature of Mg-Sn alloys was investigated by comparison of Mg-3Sn (wt.%) (T3), Mg-3Sn-1Ca (wt.%) (TX31), and Mg-3Sn-1Ca-1Zr (wt.%) (TXK311) alloys under extrusion. The results show that the main phases of as-extruded T3 alloy were α-Mg and Mg_2_Sn phases, while the CaMgSn phase was formed and the precipitation of Mg_2_Sn phase was inhibited in the TX31 and TXK311 alloys due to the addition of the Ca element. Zr did not form intermetallic compounds with other elements but dissolved in the grains of the matrix and became nucleating particles. Incomplete dynamic recrystallization (DRX) occurred in all alloys during hot extrusion. The coarse rod-like and fine block-like mixed CaMgSn phase was observed in α-Mg matrix of as-extruded samples of the TX31 alloy, and the dispersed granular CaMgSn phase was observed in the TXK311 alloy. Ca inhibited the dynamic recrystallization behavior of the alloys, while Zr promoted the dynamic recrystallization behavior. All the as-extruded alloys exhibit typical fiber texture of {0001} basal//ED. With the addition of Ca and Zr elements, the particle stimulated nucleation (PSN) effect excited by the second phase particles gradually weakened the texture. TXK311 alloy has good comprehensive mechanical properties at room temperature, with tensile strength, yield strength, and elongation of 261 MPa, 244 MPa, and 11%, respectively, and the average grain size was 1.8 μm. Grain refinement and second phase dispersion strengthening are considered to play critical roles in the strength optimization of the TXK311 alloy.

## 1. Introduction

In order to achieve the goal of “intelligence, low-carbon, and environmental protection”, the research and development of new materials has attracted much attention. Magnesium (Mg) alloys have broad application potential in electronic information, aerospace, transportation, and other fields due to their low density and meeting the needs of weight reduction [1,2]. It is known as the lightest metal structure material at present due to its high specific strength, high modulus of elasticity, good damping resistance, and other advantages [3,4]. However, the undesirable strength and plastic deformation capacity restricts further application of Mg alloys.

Mg-Sn alloys have attracted extensive attention because of their potential to improve mechanical properties. Generally speaking, adding a small amount of Sn to Mg and Mg alloys can form a Mg_2_Sn phase with high melting point [5]. The melting point of the binary phase Mg_2_Sn (772 °C) is much higher than that of the traditional Mg_17_Al_12_ phase (462 °C), and thus the Sn can be used as the strengthening phase in heat-resistant Mg alloys. Compared with Rare-earth elements, the preparation cost of Sn is low, and the Mg-Sn alloys with high melting point can be extruded in a wider range of temperatures and speeds. Thus, it is possible to develop “Rare-earth free” magnesium alloys with excellent heat resistance [6,7]. However, the production of Mg–Sn alloys using conventional ingot metallurgy results in a Mg matrix with coarse grain sizes and large dendrite arm spacing [8]. Besides, the mechanical properties of as-extruded Mg-Sn based alloys with high Sn addition (more than 8 wt.%) are still poor, and its application is limited because Sn is a relatively expensive element [9,10]. Therefore, the development of Mg-Sn alloys with cost-effective, high strength, and excellent plasticity is a difficult problem to be solved [11]. Generally, micro-alloying, grain refinement, and deformation treatment can improve the mechanical properties of Mg alloys [12,13,14]. It was reported that different extrusion speeds can affect the occurrence of dynamic recrystallization, and then affect the microstructure and mechanical properties of the alloy [15]. Zhang et al. [16] studied the effects of different extrusion ratios on the microstructure and mechanical properties of Mg-Sn-Zn-Ca alloys, and found that the dynamic recrystallization rate and average grain size of the alloy decreased with the increase of extrusion ratio, and the strength and elongation increased to varying degrees. The addition of alloying elements can improve the strength and plastic deformation ability of the alloy through the second phase strengthening. Wu et al. [17] studied the effect of Al on the microstructure and mechanical properties of Mg-Sn-Ca alloys and found that the type and content of nano-sized second phase in the alloy changed and the elongation of the alloy improved with the increase of Al content.

In recent years, in order to improve the mechanical properties of binary Mg-Sn alloys, scholars have tried to alloy different elements [18,19]. CaMgSn and Mg_2_Ca phases can be generated by adding Ca into Mg-Sn alloys, and a small amount of Ca can improve the tensile strength and elongation of Mg alloys. Zhao et al. [20] studied the microstructure, mechanics, and corrosion properties of Mg-Sn-Ca alloys and found that the strength of the alloy increased when the Ca content increased to 1.5% or the Sn content increased to 2% of the Mg-1Sn-0.5Ca alloy. Kozlov et al. [21] demonstrated that the second phase will transform with the change of Sn/Ca ratio in Mg-Sn-Ca alloys, thus affecting the mechanical properties. Suresh et al. [22] studied the effect of different Sn and Ca contents on the microstructure and mechanical properties of as-cast Mg-Sn-Ca alloys. They believed that CaMgSn phase would be preferentially formed when Sn and Ca existed at the same time, and the grains would be significantly refined to improve the microhardness when the Sn/Ca ratio was about 3. Zhang et al. [23] found that Ca is one of the most effective elements to improve the mechanical properties of the Mg-Sn alloy, and CaMgSn has the most inhibitory effect on grain coarsening, followed by Mg_2_Ca, and finally Mg_2_Sn. Moreover, in the extruded Mg-Sn-Ca alloy, CaMgSn phase can effectively maintain the tensile strength of the alloy and improve the creep resistance of the alloy at high temperature.

Zr is a common alloy element, which has a significant grain refinement effect on non-aluminum Mg alloys. It is reported that adding a small amount of Zr can effectively refine the grains of Mg-Dy-Sm alloys and improve the strength of Mg alloys [24]. Sadeddin et al. [25] found that Zr element improved the microstructure stability of the Mg-5Sn alloy during high temperature homogenization treatment. Xu et al. [26] investigated the effect of Zr addition on the microstructure and mechanical properties of as-extruded Mg-2Gd-1.2Y-0.5Zn (at.%) alloy. They revealed that the addition of Zr could effectively refine the microstructure of the homogeneous alloys and was conducive to the dissolution of the second phase. Liu et al. [27] illustrated the effect of Zr on the microstructure and mechanical properties of the AZ91 Mg alloy and found that the appropriate addition of Zr can become the heterogeneous nucleation core, which can effectively refine the grains of the AZ91 Mg alloy, improving the tensile strength and reduction of area of the alloy at room temperature.

In conclusion, as a promising Rare-earth free magnesium alloy with excellent heat resistance, the mechanical properties of Mg-Sn alloys need to be further improved, and the strength of the alloy can be effectively strengthened and the plastic deformation ability can be improved through extrusion deformation, second phase strengthening, and fine grain strengthening. Therefore, adding Ca and Zr to the extruded Mg-Sn alloy with low Sn content for microalloying is a promising choice. At present, there are relatively few studies on adding Ca and Zr to as-extruded Mg-Sn alloy. Therefore, in this study, the as-extruded T3 alloy was selected as the reference material to form the high melting point CaMgSn phase by adding 1% Ca, and at the same time, 1% Zr was added to the alloy to achieve the purpose of refining the CaMgSn phase. The effects of Ca and Zr addition on the microstructure and mechanical properties at room temperature of the as-extruded T3 alloy were systematically studied in order to improve the mechanical properties of the Mg-Sn alloy. The maximum tensile strength of 261 MPa and the yield strength of 244 MPa were obtained, respectively. The high strength of the as-extruded TXK311 alloy was composed of dispersed CaMgSn phase and ultrafine grains.

## 2. Experimental Procedure

**Figure 1:**
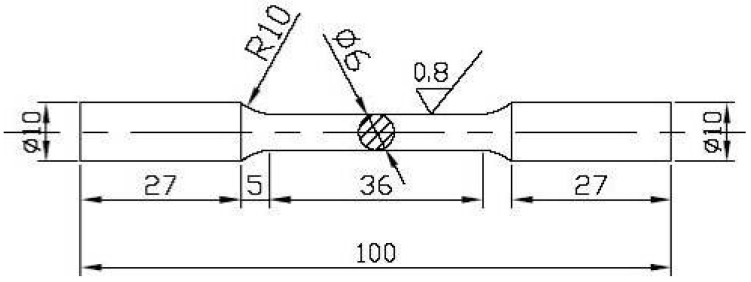
The dimensions of tensile specimens used in this study (unit: mm).

**Figure 2:**
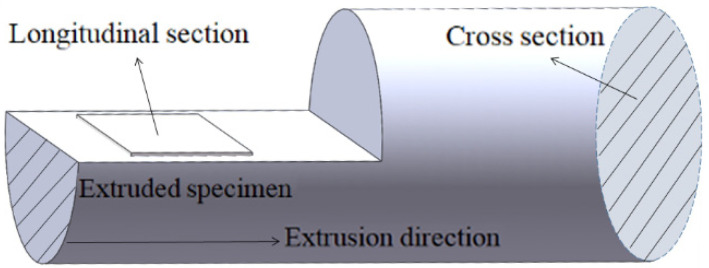
Metallographic sampling location of as-extruded alloy.

In this work, as-cast T3, TX31, and TXK311 alloys were prepared in a well type resistance furnace (RJ2 series, Hankou electric furnace company, Wuhan, China) with master alloys of industrial pure Mg (purity > 99.99%), pure Sn (99.9 wt.%), Mg-30% Zr, and Mg-25% Ca as raw materials, respectively. During melting, pure Mg should be put into a preheated crucible and heated to 750 °C. After pure Mg was completely melted, alloy elements were added under the protection of mixed gas (CO_2_: SF_6_ = 100:1). After all alloy elements were melted, fully stirred, and cooled to 710 °C for 25 min, and then the metal solution was cast into a steel mold with a diameter of 65 mm and a height of 240 mm under the protection of mixed gas, which was naturally cooled and formed. The prepared ingots were homogenized in a box type resistance furnace (1400 °C manual door box furnace, Henan Sante Furnace Technology Co., Ltd., Luoyang, China) at 400 °C for 24 h, and uniform alloy samples were obtained by water quenching and cooling. The actual components of as-homogenized samples were detected by inductively coupled plasma atomic emission spectrometer (ICP, Plasma 2000, Sinovac Testing Technology Co., Ltd., Beijing, China), and the results are shown in Table 1. The bar with diameter of 12 mm (extrusion ratio 15:1) was obtained by reverse extrusion of 300 tons vertical hydraulic press at extrusion temperature of 300 °C and extrusion speed of 1 mm/s. The tensile test was carried out at the tensile rate of 1 mm/min using the electronic universal testing machine (Wdw-100, Jinan Times gold test instrument Co., Ltd., Jinan, China) at room temperature. Each alloy sample was subjected to three groups of parallel tests (The tensile sample is processed according to GB228-87, and the sample size is shown in Figure 1). The phase of the as-extruded sample was identified by X-ray diffraction analyzer (Shimadzu—7000, Shimadzu (Hong Kong) Co., Ltd., Hong Kong, China) (the target is Cu; the experimental voltage and current were 40 kV and 30 mA respectively; the experimental scanning angle was 20–90°). The sampling position of the as-extruded alloy is shown in Figure 2. The microstructure of the as-extruded samples was characterized by scanning electron microscope (SEM) and energy dispersive spectroscopy (EDS) (Hitachi S-4800, Hitachi, Tokyo, Japan). The grain orientation information of the sample was obtained by electron backscatter diffraction (EBSD) (USA EXAX-TSL, Oxford Nordly max3, Oxford Instruments (Shanghai) Co., Ltd., Shanghai, China), and the data were analyzed by Channel 5 software.

## 3. Results and Analysis

### 3.1. Microstructure Characteristics of As-Extruded Alloy

**Figure 3:**
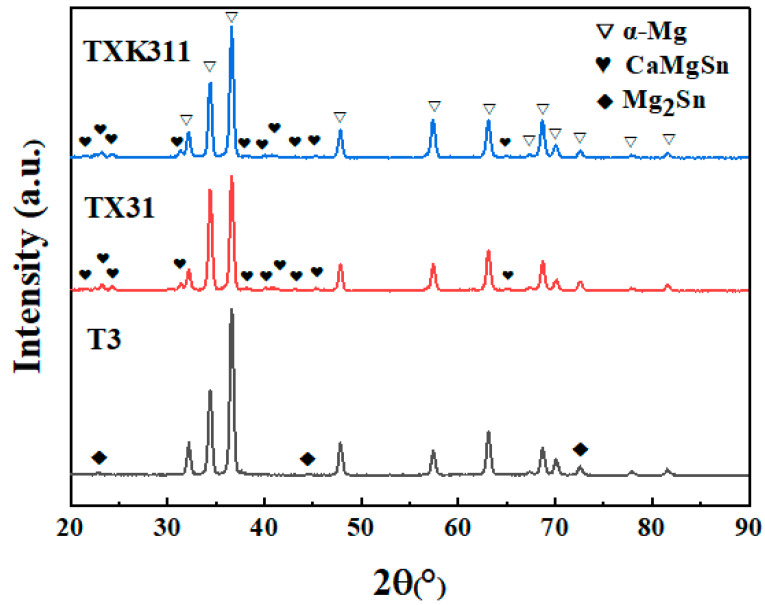
XRD patterns of as-extruded T3, TX31, and TXK311 alloys.

Figure 3 shows the XRD patterns of as-extruded T3, TX31, and TXK311 alloys. According to the phase analysis combined with the spectrum, T3 alloy is mainly composed of α-Mg and Mg_2_Sn phases. Only the diffraction peaks of α-Mg and CaMgSn phases exist in the alloy with the addition of 1% Ca alone, but the diffraction peaks of Mg_2_Sn and Mg_2_Ca phases were not detected. In the XRD pattern of the as-extruded TXK311 alloy, there was no diffraction peak containing Zr, and only the diffraction peak of α-Mg and CaMgSn phases can be found, which may be due to the solid solution of Zr in α-Mg matrix.

**Figure 4:**
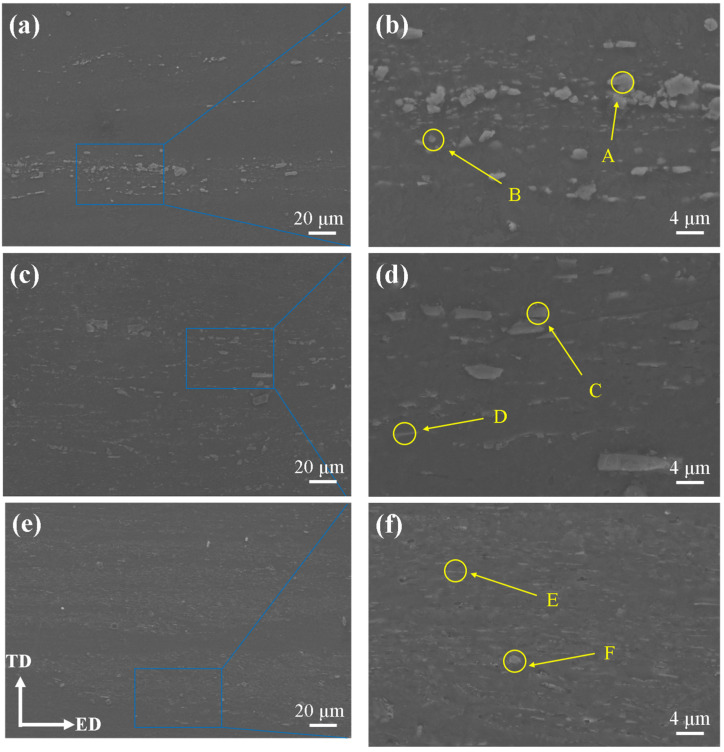
SEM of as-extruded alloys: (**a**,**b**) T3, (**c**,**d**) TX31, (**e**,**f**) TXK311. (**ED**: Extrusion direction, **TD**: Transverse direction).

In order to further determine the composition and distribution of the second phases of the alloy, the SEM images of the as-extruded T3, TX31, and TXK311 alloys were detected on the longitudinal section, as shown in Figure 4. The area fraction and size of the second stage was calculated by Image Pro Plus software, as shown in Table 2. The results show that the dynamic recrystallization of the three alloys occurred obviously during extrusion. The second phases gathered in the T3 alloy were obviously broken, with an average size of 2.4 μm and an area fraction of 2.4%. The second phases of the broken block (positions A and B) were intermittently distributed along the extrusion direction (Figure 4a). After the addition of Ca element, parts of the second phases of the alloy were broken, with an average size of 1.7 μm and an area fraction of 4.6%. The blocky and short rod-like second phases (positions C and D) after fracture (Figure 4c) present a streamline distribution along the extrusion direction. In contrast, the second phases of the TXK311 alloy were more evenly distributed, with an average size of 1.3 μm and an area fraction of 4.9%. It is mainly dispersed in the grains of the matrix in fine needle-like and granular shapes (positions E and F) (Figure 4e). Combined with the XRD spectrum in Figure 3 and the EDS results in Table 3, the massive phases in the T3 alloy were Mg_2_Sn phase distributed along the grain boundary. The Sn/Ca ratio of the short rod-like and blocky second phase in the TX31 alloy was close to 1, which was presumed to be CaMgSn phase. Compared with the T3 alloy, the change of precipitated phase was due to the addition of Ca element. It has been reported that the type and quantity of precipitates in Mg-Sn-Ca alloys were related to the atomic ratio of Sn and Ca elements in the alloy. As the mass ratio of Sn/Ca was about 3:1, almost all Ca will combine with Mg and Sn to form a stable CaMgSn phase [28]. After adding Ca and Zr elements, the Sn/Ca content ratio of fine needle-like and granular second phases in the alloy were close to 1 and Zr element was contained in the second phase. The analysis shows that the second phases of TXK311 alloy were CaMgSn phase, while Zr element was solidly dissolved in the granular phase of the matrix and became nucleating particles and does not form intermetallic compounds with other elements. Over the past few years, Liu et al. [29] also found the same phenomenon in Mg-5Sn-xZr alloys—that Zr was enriched in the matrix particle phases and became nucleation particles.

**Figure 5:**
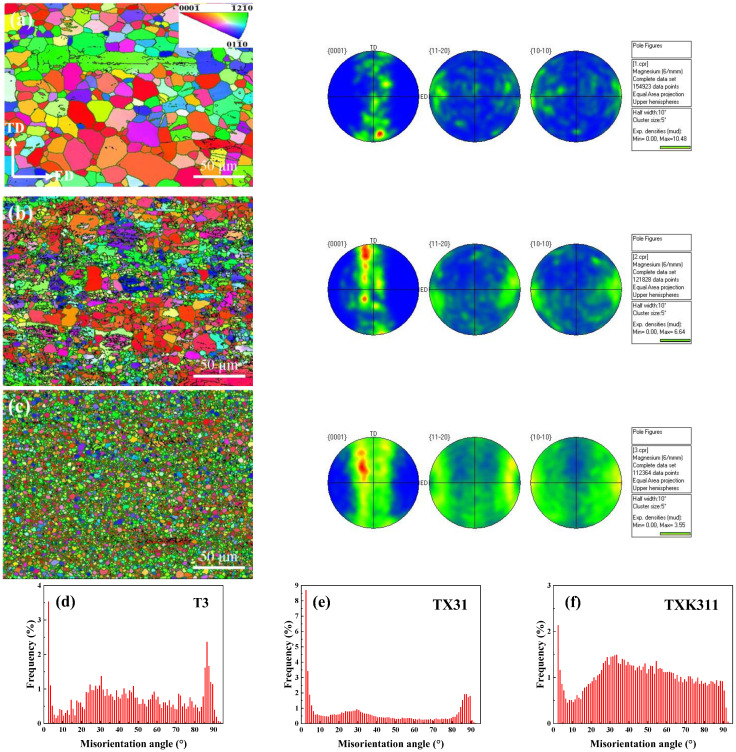
IPF and PF maps of as-extruded alloys: (**a**) T3, (**b**) TX31, (**c**) TXK311; (**d**–**f**) Misorientation angle distribution of three as-extruded alloys.

Mg alloys are prone to produce preferred texture during the forming process, and especially at room temperature, the anisotropy is more prominent [30]. Since texture has a serious impact on the plasticity of the material, in order to further study the evolution of texture and grain orientation after extrusion, we determined the inverse pole figure (IPF) maps, pole figure (PF) maps, and orientation angle distribution of the as-extruded alloys shown in Figure 5. As shown in Figure 5, the three as-extruded alloys all exist at fiber texture {0001} basal//ED, which is a typical texture of extruded Mg alloys [31]. For {0001} basal, the maximum polar density values of T3, TX31, and TXK311 alloys were 10.48, 6.64, and 3.55, respectively. In addition, it can be seen from the figure that the low angle grain boundaries (LAGBs) (black) fractions of the three alloys were 6.55%, 17.61%, and 6.87%, respectively. The high angle grain boundaries (HAGBs) (green) fractions were 60.07%, 43.86%, and 85.56%, respectively. The results showed that with the addition of 1% Ca, the texture strength of the alloy becomes weak and the orientation difference between adjacent grains decreases. However, by combining addition of 1% Ca and 1% Zr element, the texture strength of the alloy becomes weaker and the proportion of HAGBs increases significantly. It is considered that the particle stimulated nucleation (PSN) mechanism is the key factor to weaken the strength of texture. Studies have reported that coarse particles with a size greater than 1 μm can trigger PSN mechanism, reducing the orientation correlation between recrystallized grains and existing grains, and thus weakening the texture strength [32,33].

**Figure 6:**
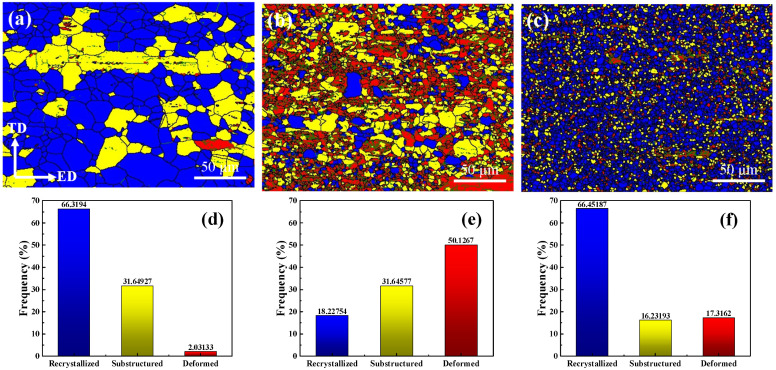
Different types of grain distribution: (**a**,**d**) T3, (**b**,**e**) TX31, (**c**,**f**) TXK311.

Figure 6 shows the EBSD diagram of different types of grain distribution of the as-extruded T3, TX31, and TXK311 alloys and the corresponding fractions of each region. Blue, yellow, and red areas represent recrystallized grains, sub-grains, and deformed grains, respectively. Studies have shown that when the extrusion temperature of Mg alloy exceeds 350 °C, there is a completely dynamic recrystallization [34,35,36,37]. Due to the extrusion temperature set in this work being 300 °C, the completely dynamic recrystallization occurred in the three alloys during extrusion. It can be seen from the figure that the recrystallized grain fraction of the T3 alloy was 66%, and the sub-grain fraction was 32%, with a small quantity of deformed grains. After adding 1% Ca alone, the microstructure of the alloy was composed of a small amount of dynamic recrystallized grains and severely elongated non-dynamic recrystallized grains. The proportion of dynamically recrystallized grains was only 18%, while the deformed grains increase to 50%. After adding 1% Ca and 1% Zr, a large amount of dynamic recrystallization occurred in the alloy. The grain fraction of dynamic recrystallization increased to 67%, while the grain fraction of sub-grain decreased to 15%, and the deformed grain fraction was 17%. The results show that the addition of alloying elements will affect the dynamic recrystallization behavior of the alloy during extrusion. Precipitates may accelerate or retard the recrystallization process depending on their size and volume fraction [38]. The fine dispersion particles block the grain boundary movement through Zener Pinning effect and slows down the recrystallization and grain growth. However, coarse particles can accelerate recrystallization through PSN because of the large amount of energy stored in the deformation region. The analysis shows that after adding 1% Ca, the second phase particles of the alloy become smaller (as shown in Figure 4), resulting in a pinning effect, which hinders the movement of grain boundaries. Moreover, the area fraction of LAGBs increases, which makes the distribution of dislocations uniform and stable during deformation, thus inhibiting nucleation and hindering the occurrence of dynamic recrystallization. Studies have shown that adding 0.4 or 0.8 wt.% Ca to Mg-6Zn alloy can inhibit the occurrence of dynamic recrystallization during extrusion [39]. After adding 1% Ca and 1% Zr, the dynamic recrystallization degree of the alloy is the highest, which can be attributed to the addition of Zr. The addition of Zr increases the HAGBs fraction in the alloy, which indicates that the crystal energy increases and the recrystallization fraction increases. At the same time, according to the EDS results in Table 3, Zr enriched in the granular phase in the matrix can become nucleating particles, which promotes the dynamic recrystallization behavior. Studies have shown that Zr has a dense hexagonal structure as close as Mg, which can serve as the core of nucleation and play the role of heterogeneous nucleation [40].

In addition, it can be seen from Figure 6 that the average grain size of the as-extruded T3, TX31 and TXK311 alloys were 9.9 μm, 3.8 μm, and 1.8 μm, respectively. This is because the precipitation of the second phase CaMgSn hinders the grain growth. According to the SEM results in Figure 4, part of CaMgSn phases were distributed inside the α-Mg grain. The CaMgSn phase preferentially generated during the solidification process can be used as the heterogeneous nucleation core of the later α-Mg, which can improve the nucleation rate of α-Mg and further refine the grain size.

Previously, Jiang et al. [41] illustrated the effect of heterogeneous nucleation and grain refinement of CaMgSn from the perspective of crystallography when studying the effect of the second phase on the microstructure and mechanical properties of as-cast Mg-Ca-Sn alloys. Compared with TX31 alloy, the grains of the TXK311 alloy were further refined into equiaxed grains, which can be attributed to the peritectic reaction of Zr and component supercooling. According to the Mg-Zr binary phase diagram, when the Zr content is greater than 0.59%, Zr will have a peritectic reaction with Mg [42]. In the peritectic reaction, β-Zr can be used as the heterogeneous nucleation core in α-Mg, and the fast growth of α-Mg can be prevented by the solid phase diffusion characteristics of the atom so as to refine the grain. Lee et al. [43] illustrated that the growth restriction factor (GRF) of Zr element in Mg alloys is 38.29, which is much higher than that of other elements. In this test, Zr content is more than 0.59%, and a large amount of β-Zr dispersed particles will be formed in the magnesium liquid, which will significantly refine the grain. A portion of Zr can become the core of inhomogeneous nucleation of α-Mg matrix, while the other part of Zr was dissolved in the matrix particle phase. During the solidification process of the alloy, the component supercooling zone can be generated to inhibit the grain growth, and the dual effect makes the grain refinement more obvious. Furthermore, Liu et al. [29] discussed the mechanism of adding Zr element to refine the grain of Mg-5Sn alloy. They believe that Zr element can refine the alloy structure through the dual effects of forming heterogeneous nucleation particles when Mg and Zr undergo peritectic reaction at 645 °C, inhibiting the grain growth of Zr due to component supercooling.

### 3.2. Mechanical Property Analysis

**Figure 7:**
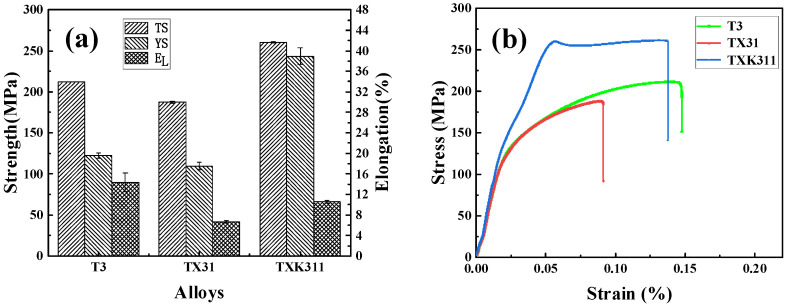
Mechanical properties of as-extruded T3, TX31, and TXK311 alloys at room temperature: (**a**) Mechanical properties diagram, (**b**) Engineering tensile stress–strain curves.

Figure 7 shows the engineering stress–strain curves, mechanical properties, and strain hardening index of as-extruded T3, TX31, and TXK311 alloys at room temperature. It can be seen that the tensile strength, yield strength, and elongation of the T3 alloy at room temperature were 212 ± 0.1 MPa, 123 ± 3.4 MPa, and 15 ± 1.9%, respectively. After adding 1% Ca alone, the mechanical properties of the alloy showed a downward trend, the tensile strength and yield strength decreased to 188 ± 1.2 MPa and 110 ± 4.3 MPa, respectively, and the elongation of the alloy was only 7 ± 0.3%. When adding 1% Ca and 1% Zr, the tensile strength and yield strength of the alloy were 261 ± 0.8 MPa and 244 ± 10.3 MPa, respectively. However, the plastic deformation ability of the alloy was not improved, and the elongation was 11 ± 0.3%.

Generally speaking, grain size, second phase, texture, or grain orientation are important factors that affect the yield strength and ductility of Mg alloys [44]. Research shows that grain refinement will lead to the increase of grain boundaries, making the transmission of dislocations difficult, thus improving the strength of the alloy [45]. According to Hall Petch formula, the yield strength of materials is expressed as a function of grain size, as shown in Formula (1) [46].

(1) $$\sigma_{s}=\sigma_{0}+Kd^{-1/2}$$

where: $\sigma_{s}$ is the yield strength, $\sigma_{0}$ is the friction stress, *K* is the constant, and *d* is the average grain diameter. Obviously, the yield strength is inversely proportional to the grain size, and fine grains can improve the yield strength of the alloy. Assume that all as-extruded alloys in this study $\sigma_{0}$ is the same as *K* value, then the increase in yield strength caused by grain refinement can be expressed as:

(2) $$\sigma_{grain}=Kd^{-1/2}$$

At present, studies have found that the *K* value of as-extruded alloy is about 170 MPa µm^−1/2^ [47]. The average grain size of the three as-extruded alloys were 9.9 μm, 3.8 μm, and 1.8 μm, respectively. Therefore, compared with T3 alloy, the yield strength increment of TX31 and TXK311 alloy caused by grain refinement was 87.21 MPa and 126.71 MPa, respectively. The calculated yield strength is very close to the measured yield strength. It is indicated that the main factor for the increase of the yield strength of TXK311 alloy is the fine grain strengthening effect, and the reason for the decrease of the measured strength of TX31 alloy may be due to the effect of the second phase.

Zeng et al. [48] found that the second phase plays an important factor affecting the strength of Mg alloys. The morphology, distribution, and volume fraction of the second phase, especially brittleness or toughness, affect the strength and ductility of Mg alloys. It is assumed that the second phase particle enhancement mechanism of these three alloys follows the Orowan equation [49]. The specific calculation basis is as follows:

(3) $$YS\propto f^{1/2}D^{-1}InD$$

where: *D* is the average diameter and *f* is the volume fraction of the second phase. Obviously, reducing the average diameter of the second phase particles and increasing the area fraction of the second phase can improve the yield strength of the alloy. In this work, with the addition of 1% Ca and 1% Zr, the amount of second phase precipitation increases (see Figure 4), which helps to improve the yield strength of the alloy. In addition, it has been reported that the fine second phase interacts with dislocation to hinder dislocation movement, thus improving the strength of the alloy, while the brittle second phase dispersed in a-Mg matrix breaks first under tensile stress, leading to a sharp decline in the plasticity of Mg alloys [50]. In TX31 alloy, the addition of 1% Ca transformed the second phase of the alloy from massive Mg_2_Sn to rod-shaped brittle CaMgSn precipitated at the grain boundary. Therefore, under the action of tensile stress, CaMgSn phase will have a splitting effect on the matrix, and at the same time, the plastic deformation of the alloy will be uneven, resulting in stress concentration, which will promote the formation of cracks and lead to the deterioration of the mechanical properties of the alloy. Compared with TX31 alloy, the addition of 1% Zr in TXK311 alloy reduces the amount of quantity CaMgSn precipitation at the grain boundary, and the granular CaMgSn phase disperses and distributes in the matrix grains, forming the second phase dispersion strengthening, which improves the ability to hinder grain boundary sliding and dislocation movement, thus improving the mechanical properties of the alloy.

**Figure 8:**
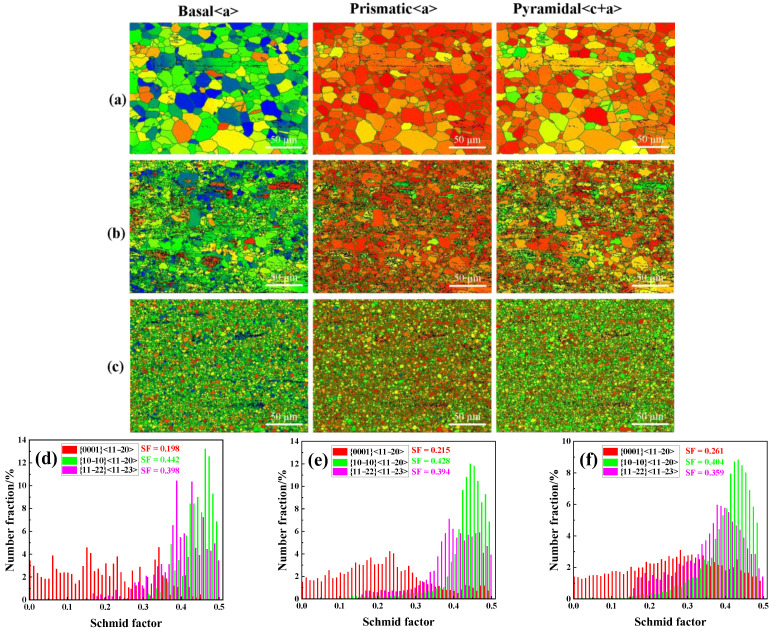
Distribution of Schmid factors: (**a**,**d**) T3, (**b**,**e**) TX31, (**c**,**f**) TXK311.

Studies have shown that in the process of metal plastic deformation, Schmidt Factor (SF) can qualitatively analyze the starting trend of various dislocation slip mechanisms, and the starting of slip usually follows Schmidt’s law [51]. In order to study the main deformation mechanism of the three as-extruded alloys in this test, the SF diagrams of basal <a> slip, prismatic <a> slip, and pyramidal <c+a> slip shown in Figure 8 were determined. It can be seen from Figure 8 that the average SF value of basal <a> slip of the three alloys was lower than that of prismatic <a> slip and pyramidal <c+a> slip. Therefore, the main deformation mode of the three as-extruded alloys was prismatic <a> slip, supplemented by pyramidal <c+a> slip. In addition, with the increase of 1% Ca and 1% Zr, the basal <a> slip SF increases significantly, and the difference of SF along different directions decreases, resulting in the improvement of ductility and the weakening of anisotropy of the alloys. However, the test results showed that T3 alloy has the best plasticity and TX31 alloy has the worst plasticity. The main reason is that the brittle phase CaMgSn in the alloy is prone to stress concentration, which becomes the source of cracks and leads to the decline of mechanical properties of the alloy.

**Figure 9:**
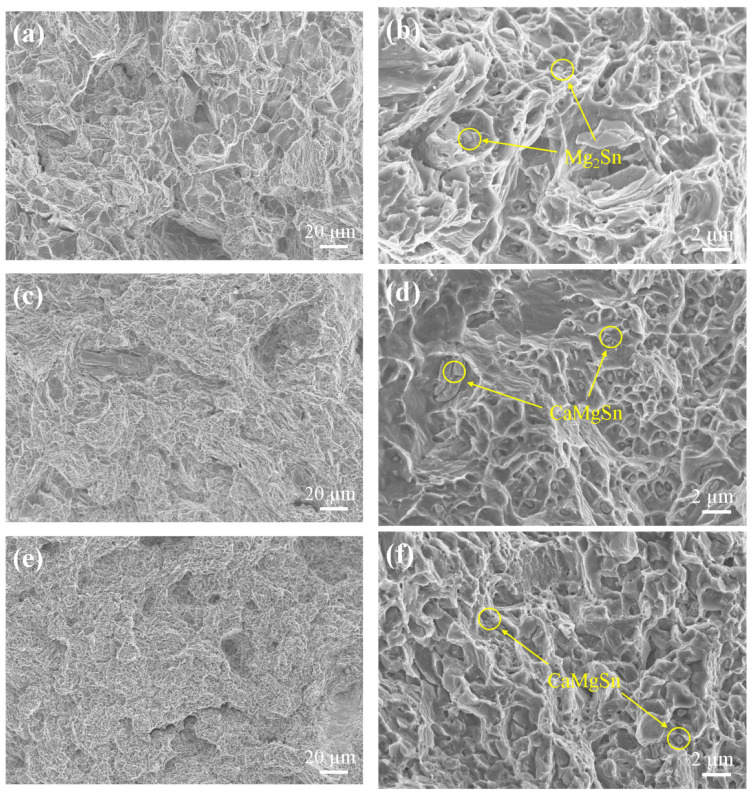
SEM fracture morphology of as-extruded alloys tensile fracture specimen: (**a**,**b**) T3, (**c**,**d**) TX31, (**e**,**f**) TXK311 ((**b**,**d**,**f**) are partial enlarged views of (**a**,**c**,**e**)).

In order to further study the influence of the fracture mechanism of the alloy on the mechanical properties, Figure 9 shows the SEM fracture morphology of the tensile fracture specimen of the alloy after extrusion. It can be seen from the figure that there were a large number of dimples and inclusions on the fracture surfaces of the three alloys. Dimples are generally considered as the symbol of the ductile fracture mode, indicating that the main failure mode of the three alloys is ductile fracture [52]. The EDS results showed that the inclusions in the dimple of T3 alloy were Mg_2_Sn phase, the inclusions in the TX31 and the TXK311 alloys were CaMgSn phase, and the inclusions in the TX31 alloy were the most. In addition, the fracture morphology of the T3 alloy were dark and fibrous, and there are a large number of thick and deep dimples. There are obvious tearing edges at the fracture, which indicates that the T3 alloy has good plasticity. However, in the TX31 and the TXK311 alloys, dimples were small and deep, and cleavage planes exist in the fracture, which was considered to be cleavage fracture, and was a typical mixed fracture of toughness and brittleness. It can be seen from Figure 7a that the plasticity of the TX31 alloy was the worst and that of the T3 alloy was the best. Based on the analysis of Figure 9, it is believed that the CaMgSn phase found in the dimple is the main reason for the decrease of the plasticity of the alloys. Wang et al. [50] found that the large brittle second phase will lead to the decrease of the plasticity of the magnesium alloy. Under tensile stress, stress concentration occurs at the interface between the Mg matrix and the second phase CaMgSn particles, which promotes the formation of cracks. With the continuation of deformation, cracks in local areas merge and tear along the edge, thus reducing the mechanical properties of the alloy at room temperature. It can be concluded that the addition of Ca and Zr elements to refine the grains is the main reason for the improvement of the mechanical properties of the alloy in this study, but the brittle second phases will have a negative impact on the mechanical properties of the alloy. The mechanical properties of the alloy at room temperature can be improved by refining the second phase particles to produce the second phase dispersion strengthening effect.

**Figure 10:**
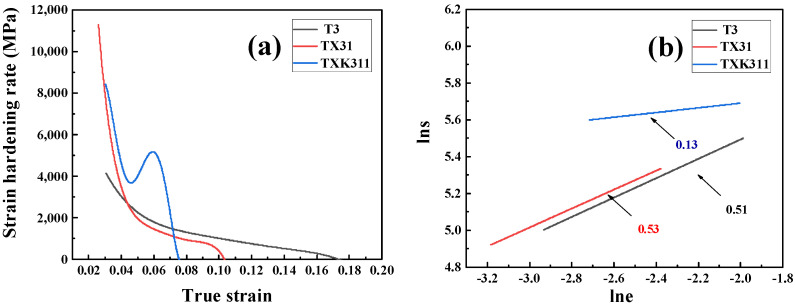
Strain hardening rate (**a**) and strain hardening index (**b**) of three as-extruded alloys.

In order to analyze the strain hardening behavior of three as-extruded alloys, the strain hardening rate versus true strain curve and strain hardening index curve were drawn, as shown in Figure 10. The work hardening rate is derived as follows [53]:

(4) $$\theta =(\frac{\delta \sigma }{\delta \varepsilon }{)}_{\dot{\varepsilon }}$$

where δσ, δε, and $\dot{\varepsilon }$ are the true stress, true strain, and strain rate, respectively. In Figure 10a, the strain hardening rate of the three as-extruded alloys from high to low was TX31 > TXK311 > T3. The higher strain hardening rate of the TX31 alloy may be due to the high strain mismatch or incompatibility between the second phase and α-Mg matrix. Due to the existence of brittle phase CaMgSn in the alloy, it is easy to produce stress concentration and accumulate more dislocations, thus improving the strain hardening ability [54]. The TXK311 alloy was due to the high area fraction of dynamic recrystallization, resulting in low defect density in grains, that is, the formation of high fraction of HAGBs, which can more effectively promote uniform deformation. The mechanism is that the HAGBs can more effectively prevent dislocation movement, promote dislocation aggregation and entanglement around the grain boundary, resulting in high strain hardening rate [55]. Figure 10b shows the strain hardening index of three as-extruded alloys. According to Figure 7b, TXK311 alloy has the lowest strain hardening index, while T3 and TX31 alloys have little difference in strain hardening index. The results show that the curve of the TXK311 work hardening section was relatively stable, indicating that the alloy has little resistance to deformation, weak local strain ability, and is easy to enter dispersion instability, that is, the uniform deformation ability of the alloy is poor. It shows that the addition of Ca element is conducive to improve the work hardening ability of the alloy, while the addition of Zr element is not conducive to the improvement of strain hardening ability.

Finally, the feasibility and limitations of the existing samples are discussed from the perspective of cost-effectiveness and mechanical properties. The tensile strength, yield strength, and elongation of the TXK311 alloy at room temperature were 261 MPa, 244 MPa, and 11%, respectively, and the average grain size decreased to 1.8 μm. The texture strength decreased to 3.55. Compared with the current commercial Mg alloys, the yield strength of the alloy is higher than that of the as-extruded bar AZ31B (140 MPa) and AZ91D (160 MPa), which can be comparable to the newly developed Mg-3Nd-0.2 Zn-0.5 Zr (200 MPa) [56] and Mg-5Zn-8Y-0.6Zr (256 MPa) [57]. It has potential application value in automobile parts, machine parts housing, and communication equipment. In addition, the addition amount of Sn and cheap elements Ca and Zr is limited, which saves cost-effectiveness. In addition, the addition amount of Sn and cheap elements Ca and Zr is limited, which saves cost-effectiveness. Compared with Rare-earth materials, TXK311 alloy has poor plastic deformation ability. Therefore, the mechanical properties of existing alloys still need to be further improved, and its wide application can be promoted by microalloying.

## 4. Conclusions

Mg-Sn alloy is an important “Rare-earth free” wrought Mg alloy. In order to improve its strength, the effects of Ca and Zr elements on the microstructure and room temperature mechanical properties of as-extruded Mg-3Sn alloy were systematically studied in this paper, and the main conclusions are listed as follows:

1. The addition of 1 wt.% Ca alone and 1 wt.% Ca and 1 wt.% Zr in as-extruded Mg-3Sn alloy will lead to grain refinement, and the average grain size will be finer after composite addition.
2. The addition of Ca makes the second phase of Mg-3Sn-1Ca and Mg-3Sn-1Ca-1Zr alloys change from Mg_2_Sn phase to CaMgSn phase. The addition of Zr makes the second phase of the alloy disperse and distribute in the grains of the matrix. The texture of Mg-3Sn alloy was weakened by PSN mechanism.
3. In Mg-3Sn alloy, Ca elements can inhibit the dynamic recrystallization behavior of the alloy through the pinning effect of grain boundary. In Mg-3Sn-1Ca-1Zr alloy, Zr element can become dispersed particles, provide nucleation points for dynamic recrystallization, and improve nucleation rates.
4. The high strength of Mg-3Sn-1Ca-1Zr alloy is due to the synergistic effect of finer grain size and second phase dispersion distribution. The poor mechanical properties of Mg-3Sn-1Ca alloy are caused by the brittle phase CaMgSn with large diameter.

## Figures and Tables

**Table 1:** Actual chemical composition of as-extruded alloy.

		Measured Composition (wt.%)		
Alloy	Mg	Sn	Ca	Zr
T3	Bal.	2.97	0	0
TX31	Bal.	3.14	1.02	0
TXK311	Bal.	3.09	1.04	0.6

**Table 2:** Area fraction and size distribution of the second phase.

Alloy		Second Phase Size Distribution (μm)	
	Area Fraction of Second Phase (%)	Mg_2_Sn	CaMgSn
T3	2.4 ± 0.2	2.4 ± 0.6	0
TX31	4.6 ± 0.4	0	1.7 ± 0.4
TXK311	4.9 ± 0.5	0	1.3 ± 0.3

**Table 3:** EDS results of as-extruded T3, TX31, and TXK311 alloys.

Point	Chemical Composition (at.%)				Phase Types
	Mg	Sn	Ca	Zr	
A	89.15	10.85	0	0	Mg_2_Sn
B	90.74	9.26	0	0	Mg_2_Sn
C	92.75	2.77	4.48	0	CaMgSn
D	57.19	19.82	22.98	0	CaMgSn
E	99.30	0.12	0.28	0.30	CaMgSn
F	99.17	0.37	0.44	0.03	CaMgSn

## Data Availability

Not applicable.
